# Physiological adaptations to sugar‐mimic alkaloids: Insights from *Bombyx mori* for long‐term adaption and short‐term response

**DOI:** 10.1002/ece3.6574

**Published:** 2020-08-26

**Authors:** Shunze Jia, Yinghui Li, Xiangping Dai, Xiaotong Li, Yanyan Zhou, Yusong Xu, Huabing Wang

**Affiliations:** ^1^ College of Animal Sciences Zhejiang University Hangzhou China

**Keywords:** adaptation, chemical defense, ecology, insect, plant–insect interaction

## Abstract

Insects evolved adaptive plasticity to minimize the effects of the chemical defenses of their host plants. Nevertheless, the expressional response and adaptation of phytophagous specialists for long‐term adaption and short‐term response to host phytochemicals remains largely unexplored. The mulberry (*Morus alba*)–silkworm (*Bombyx mori*) interaction is an old and well‐known model of plant–insect interaction. In this study, we examined the long‐term adaption and short‐term response of the mulberry‐specialist silkworm to two sugar‐mimic alkaloids in mulberry: the commonly encountered 1‐deoxynojirimycin (1‐DNJ) and occasionally encountered 1,4‐dideoxy‐1,4‐imino‐D‐arabinitol (D‐AB1), respectively. Global transcriptional patterns revealed that the physiological responses induced by the selective expression of genes involved in manifold cellular processes, including detoxification networks, canonical digestion processes, target enzymes, and other fundamental physiological processes, were crucial for regulating metabolic homeostasis. Comparative network analysis of the effects of exposure to D‐AB1 and 1‐DNJ supported the contention that *B. mori* produced similar and specific trajectories of changed gene expression in response to different sugar‐mimic alkaloids. D‐AB1 elicited a substantial proportion of downregulated genes relating to carbohydrate metabolism, catabolic process, lipid metabolism, and glycan biosynthesis and metabolism. This study dramatically expands our knowledge of the physiological adaptations to dietary sugar‐mimic alkaloid intake and uncovered both metabolic evolutionarily responses and unique adaptive mechanisms previously unknown in insects.

## Graphical abstract

Insects evolved adaptive plasticity to minimize the effects of the chemical defenses of their host plants. In this study, we found that the transcriptional plasticity in genes related to metabolism, detoxification, digestion, and general cellular processes played a key role in both long‐term adaptation and short‐term responses of the mulberry‐specialist insect to changing sugar‐mimic alkaloids.

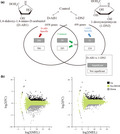

## 1 INTRODUCTION

The plant–insect interaction is among the most common and consequential ecological associations on the planet and is a key model for co‐evolution. The evolutionary histories of plant and insect diversification are closely intertwined and have served as a model in the study of co‐evolution (Futuyma & Agrawal, 2009). Plants have developed multiple strategies to combat herbivore attack, including the mobilization of specific defenses (Lortzing et al., 2017). Morphological features and the chemical composition of the plant are among the most prominent plant defenses and have long been recognized as constitutive resistance traits (Clauss, Dietel, Schubert, & Mitchell‐Olds, 2006; War et al., 2012). Plant chemicals play a critical role in defense against herbivorous insects and are crucial components of host defense generally (Gatehouse, 2002; Howe & Jander, 2008). In response to exposure to such chemically challenging or toxic environments, herbivorous have developed counter measures to against host plant toxicity and chemical defense (Després, David, & Gallet, 2007). For herbivores, gene expression plasticity could play an indispensable role in their adaptation to the various phytochemicals of host plants, enabling increased detoxification, and even reducing the amount of plant defense compounds (Dermauw et al., 2013; Wybouw et al., 2015). Apart from general detoxification enzymes, proteins with other important physiological functions are needed by herbivores to deal with host plant‐specific chemical defenses (Müller, Vogel, & Heckel, 2017). However, the expressional response and adaptive evolution of the fundamental regulators in phytophagous insects, which enable short‐term encounters and long‐term coexistence phytochemicals remain largely unexplored.

The arms race between herbivores and host plant is a classic representation of ongoing co‐evolution, which persists over long periods of evolution (Heidel‐Fischer & Vogel, 2015; Pentzold, Zagrobelny, Rook, & Bak, 2014). Herbivorous insects show various degrees of host specialization due to the need to tailor adaptive physiological features to the specific environment (Behmer, 2008; Beran et al., 2014; Dobler, Dalla, Wagschal, & Agrawal, 2012; Heidel‐Fischer & Vogel, 2015). The mulberry (*Morus alba*)–*Bombyx mori* interaction is an old and well‐known model for studying the co‐evolution of insect adaptation in response to plant chemistry. Mulberry, belonging to the Moraceae family, has been used as the sole food source for raising *B. mori* for thousands of years (Kimura et al., 2004; Konno et al., 2006). 1‐deoxynojirimycin (1‐DNJ) is a common component of mulberry leaves and occurs in higher concentrations in mulberry compared with other plants. It reduces disaccharide and the probability of α‐glucosidase binding, thereby inhibiting the decomposition of disaccharide into glucose (Hirayama, Konno, Wasano, & Nakamura, 2007). In addition, several mulberry species contain not only the main functional molecule 1‐DNJ but also other sugar‐mimic alkaloids, such as 1,4‐dideoxy‐1,4‐imino‐D‐arabinitol (D‐AB1) and 1,4‐dideoxy‐1,4‐imino‐D‐ribitol. However, the concentration of D‐AB1 varies remarkably between mulberry populations and cultivars. D‐AB1 is a potent inhibitor of glycogen phosphorylase and synthase activity, and it appears to exert adverse effects on insects by interfering with sugar metabolism in ways that are similar to their antidiabetic activities (Asano et al., 2001; Diaz‐Lobo et al., 2016). Thus, mulberry‐specialist insects need to have the physiological capacity to deal with changing levels of sugar‐mimic alkaloids. Previous studies show that the mulberry‐specialist insects, such as *B. mori*, *Bombyx mandarina*, *Glyphodes pyloalis*, and *Rondotia menciana*, were insensitive to these sugar‐mimic alkaloids (Hirayama et al., 2007). Our previous studies demonstrated that both plasticity in gene expression and evolutionary changes have extensively shaped sugar‐mimic alkaloid adaptation of nondigestive glucosidase in lepidopteran mulberry‐specialist insects (Li et al., 2018). However, how mulberry‐specialist insects evolved a systemic adaptive plasticity to cope with both long‐term and sporadic exposures to sugar‐mimic alkaloids is an ecological and evolutionary question that remains unresolved (Petschenka & Agrawal, 2016; Züst, Agrawal, & Lee, 2016). A few recent studies on the responses of the global herbivore transcriptome revealed that a substantial proportion of the transcripts show a plastic response to chemical defenses of host plants (De Panis et al., 2016; Müller et al., 2017; Ragland et al., 2015; Roy et al., 2016). Although sugar‐mimic alkaloids are among the most dominant chemicals present in mulberry latex, to date, a gene expression profile of a mulberry‐specialist species feeding on its alkaloid‐rich host plant has not yet been described.

To illuminate the expression response of insects to long‐term and sporadic exposures to sugar‐mimic alkaloids, we examined the transcriptional response of a mulberry‐specialist insect to two sugar‐mimic alkaloids of the host plant. Our results demonstrated that oligophagous insects have responded to the sugar‐mimic alkaloids of mulberry by extensively controlling the upregulation of detoxification genes and target genes of sugar‐mimic alkaloid inhibition, which enabled mulberry‐specialists to overcome development disorders in their alkaloid‐rich diet. These results deepen our understanding of the mechanism of countering host defense compounds in plant oligophagous insects. Consequently, this study allows the first insights into the diverse genes and mechanisms that are employed by insect specialists to enable their tolerance of variable plant secondary metabolites.

## 2 MATERIALS AND METHODS

### 2.1 Insects rearing and experimental setup

*Bombyx mori* (“N4 strains”) were reared at 25°C under a 12 hr light‐12 hr dark photoperiod. To investigate the effect of different sugar‐mimic alkaloids to mulberry‐specialists, newly hatched *B. mori* larvae were selected and separated into three groups with 30 larvae, respectively. In order to reduce the involvement of sugar‐mimic alkaloids, we cut the mulberry leaves into small pieces with scissors, and these strips were washed in water. The leaves were patted dry and the silkworm was fed on these leaves served as controls. The larvae were fed with 1.5 g leaves which contained 100 μl of 2% D‐AB1 and 1‐DNJ (Santa Cruz Biotechnology) as treatment group (Konno et al., 2006).

### 2.2 RNA isolation and RNA‐sequencing library preparation

For the RNA‐seq analysis, silkworms were collected and washed three times with precool phosphate‐buffered saline (PBS) after 48 hr of D‐AB1 and 1‐DNJ husbandry. Then, we ground bodies into powder with liquid nitrogen assistant, and average powder from each of the three treatment replicates per treat. We pulverized 20–30 mg of sample per individual and total RNA was extracted using RNA‐TRIzol reagent (Invitrogen) according to the manufacturer's protocol. Genomic DNA was removed from total RNA by RNase‐free DNase I (Takara). Total RNA concentration and purity were checked with a MULTISKAN GO (Thermo Fisher). The cDNA library preparation was conducted with 1 μg of total RNA using the Prime Script kit (Promega). Three biological replications were performed with each treatment.

### 2.3 Transcriptome sequencing

Sequencing was performed on BGISEQ‐500 sequencer. Raw RNA‐seq reads from the laboratory data sets were trimmed to remove primer dimers and low‐quality bases using the program SOAP nuke and Trimmomatic (Cock, Fields, Goto, Heuer, & Rice, 2010). Trimmed reads were then mapped to the *B. mori* reference genome (version 1.0; GenBank Accession Number: 7091). Mapping was conducted using Bowtie2 (Langmead & Salzberg, 2012). Then, we calculated the gene expression level by RSEM software (http://deweylab.biostat.wisc.edu/RSEM). The fragments per kilobase of transcript permillion (FPKM) were used to quantify the expression level. Principal component (PCA) analysis of FPKM was conducted using princomp function in R. To test the significance of statistics, we used the DEGseq software packages (Wang, Feng, Wang, Wang, & Zhang, 2010) with a false discovery rate of <0.001. Furthermore, false discovery rate (FDR) <0.001 and |log_2_ (fold change) | > 1.0 were used as the parameters for confirming significant differences in gene expression.

### 2.4 Gene annotation

All differentially expressed genes were functionally annotated against databases, including NCBI nonredundant protein database (Nr), Kyoto Encyclopedia of Genes and Genomes (KEGG) and Gene Ontology (GO). Enrichment analysis was calculated using clusterProfiler (Yu, Wang, Han, & He, 2012). Usually, terms with *p* < .05 was considered as significant enrichment. Term enrichment was assessed for three components of Gene Ontology: biological process (BP), molecular function (MF), and cellular component (CC). The R packages ComplexHeatmaps were used for the visualization of the differential expression analysis results (Gu, Eils, & Schlesner, 2016).

### 2.5 Investigation of temporal patterns via high‐throughput qPCR

We evaluated the temporal expression pattern profiles of candidate genes. RNA was isolated at 12, 24, 36, and 48 hr after D‐AB1 and 1‐DNJ treatment by RNA‐TRIzol reagent (Invitrogen) according to the manufacturer's protocol. Specific primers were listed in Table S1. The Prime Script kit (Promega) was used to synthesize first cDNA for 10 μl reactions according to manufacturer`s instructions. qPCR assays were performed on a Bio‐Rad CFX96 instrument. The cycling parameters were used as follows: 95°C for 30 s, followed by 40 cycles at 95°C for 15 s and 60°C for 31 s. Melting curves of the product confirmed specificity of the primers. Measured C_q_ values were analyzed using the Bio‐Rad CFX Manager System (version 3.1). The coding gene of ribosomal protein 49 (rpl49) was used as an internal control as described previously (Kiuchi et al., 2014; Zhou et al., 2019), and the ^∆∆^Ct method was used to analyze the relative differences in transcript levels. Four biological replicates and three technical replicates were performed for each sample.

## 3 RESULTS

### 3.1 Magnitude of the transcriptional regulation by D‐AB1 and 1‐DNJ

**Figure 1:**
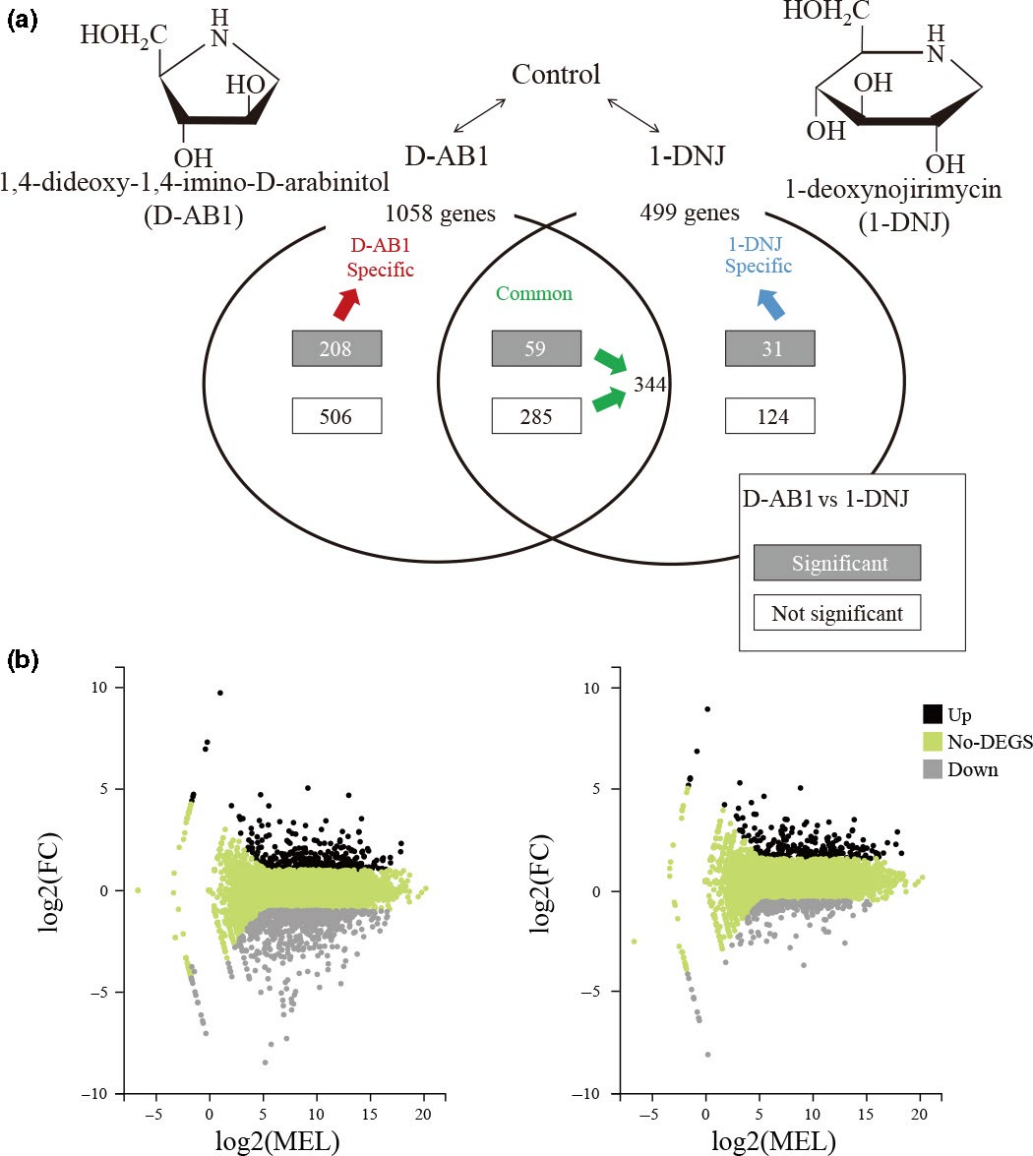
Number of differentially regulated genes in response to D‐AB1 and 1‐DNJ. (a) Numbers of DEGs (|FC| > 2 and FDR < 0.001) between untreated control and D‐AB1 or 1‐DNJ for 48 hr. Genes that were differentially regulated when comparing D‐AB1 to untreated control were further compared with those in 1‐DNJ and classified into (i) genes that were differentially regulated in both treatments when compared to untreated control (common), (ii) genes that were expressed significantly differently in D‐AB1 only (D‐AB1 specific), and (iii) genes that were expressed significantly differently in 1‐DNJ only (1‐DNJ specific). Regulation of genes when silkworms were fed on D‐AB1 that significantly differed from the 1‐DNJ treatment (DAB vs. 1‐DNJ). In D‐AB1 versus 1‐DNJ, DEGs that were also present in the untreated control compared with D‐AB1 or 1‐DNJ were marked as significant, otherwise, they were marked as not significant. For the Venn diagram displaying all of the summarized comparisons and more details on the classification, see Figure S3. (b) MA plot displaying DEGs identified in *B. mori* feeding on D‐AB1 (left) or 1‐DNJ (right) over 48 hr. Mean expression level (MEL) and fold change (FC) converted by log2 are represented by the *x*‐ and *y*‐axis, respectively. Upregulation (FC > 2) is highlighted in black, and downregulation (FC < 2) is highlighted in gray

The confrontation of herbivorous insects with an arsenal of constitutive and inducible phytochemical plant defenses is a multidimensional process. To develop a comprehensive overview of the transcriptional reaction of *B. mori* toward diverse sugar‐mimic alkaloids, we compared the transcriptomes of two sugar‐mimic alkaloids treatments against controls (i.e., D‐AB1 vs. control; 1‐DNJ vs. control). Moreover, in order to reveal the differences between the two different sugar‐mimic alkaloids, we also compared the transcriptomes from the two alkaloid treatments (D‐AB1 vs. 1‐DNJ). Our PCA results showed a clear spatial separation of the gene expression profiles for the control, D‐AB1 and 1‐DNJ treatments (Figure S1). When considering |fold change| > 2 and FDR < 0.001, 1,557 differentially expressed genes (DEGs) were found in the two alkaloid treatments D‐AB1 and 1‐DNJ. Among the 1,557 DEGs, there were 1,058 and 499 genes were differentially expressed in response to D‐AB1 and 1‐DNJ, respectively (Figure 1a,b). Furthermore, 344 were differentially expressed in both two sugar‐mimic alkaloid treatments (common), while 714 (D‐AB1 specific) and 155 (1‐DNJ specific) were only differentially expressed in D‐AB1 and 1‐DNJ treatments, respectively. Feeding on D‐AB1 led to significant changes in twice as many genes compared with the 1‐DNJ treatment. The D‐AB1 treatment significantly elicited more downregulated genes, with two‐thirds of DEGs in the common response were downregulated compared with the control larvae. In contrast, about half of the significantly modified genes were upregulated (47.70%, 238/499), and the other half were downregulated (52.30%, 261/499) in the 1‐DNJ treatment (Figure S2).

To further assess the differential effects of sugar‐mimic alkaloids, we compared the DEGs between the D‐AB1 and 1‐DNJ treatments. The analysis revealed that 337 genes were differentially expressed in total, of which 267 and 90 overlapped with the DEGs of two alkaloid treatments compared with the control larvae, respectively (Figure S3). In addition, among these 267 and 90 DEGs, 208 and 31 were only present in either D‐AB1 or 1‐DNJ, respectively (Figure 1a). Specifically, the number of DEGs identified in the D‐AB1 treatment versus the control was markedly higher than the number of DEGs detected in the 1‐DNJ treatment. A substantial alteration in the expression levels of *B. mori* transcripts after the D‐AB1 treatment may indicate particularly strong challenges when dealing with D‐AB1 exposure.

### 3.2 Functional annotation of the transcriptional response to D‐AB1 and 1‐DNJ

**Figure 2:**
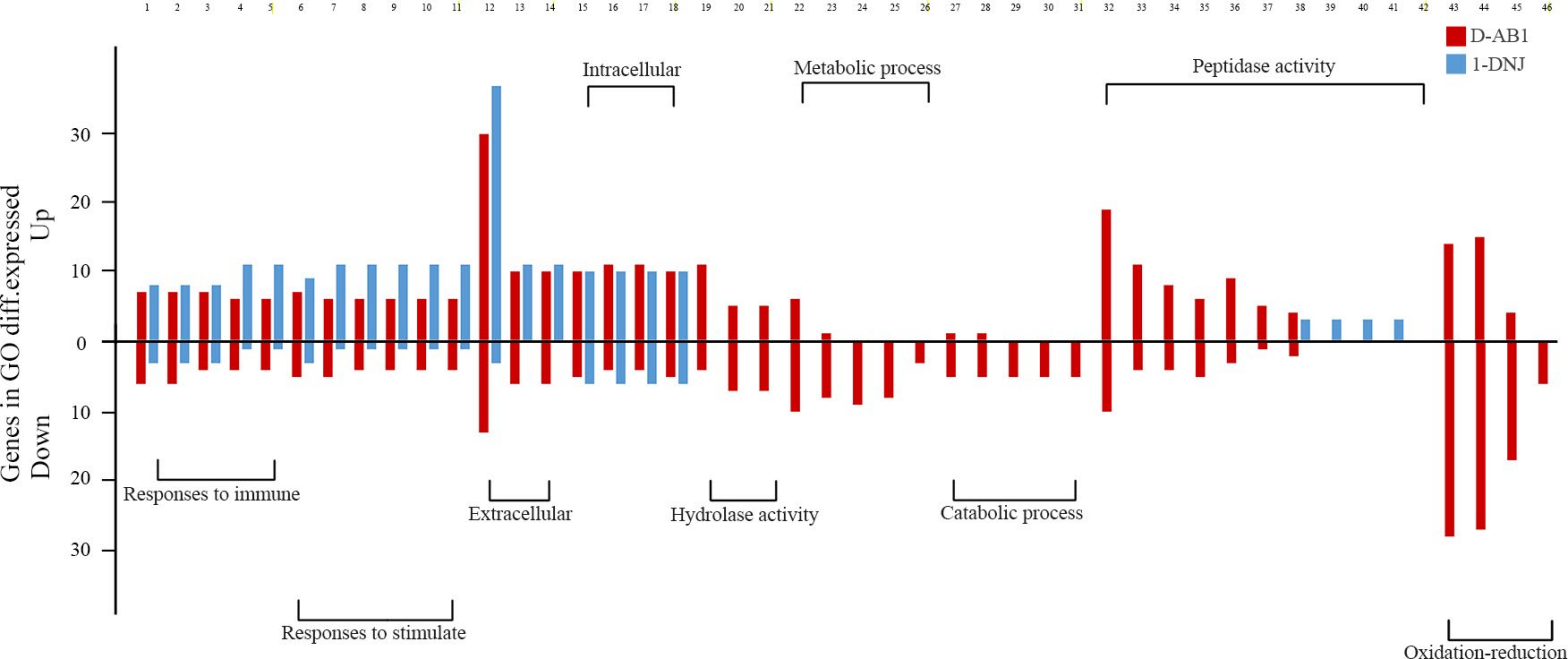
Sugar‐mimic alkaloids lead to an up‐ and downregulation of phytochemicals ‐responsive genes within enriched GO terms. Red/blue bars represent the numbers of genes that were significantly up‐ and downregulated in response to 48 hr of feeding on sugar‐mimic alkaloids. 57 GO terms were identified and are described in Table 1

To investigate the functional significance of the DEGs in each treatment, GO enrichment analysis of all annotated transcripts were executed. Genes with a significant effect (|fold change| > 2, FDR < 0.001) were significantly enriched for 56 GO terms in the D‐AB1 treatment (Table S2). GO term enrichment analysis also revealed 33 highly enriched GO terms for the set of genes that responded to 1‐DNJ (Table S3). In both treatments, several typical stimulate‐induced GO terms were identified, such as “response to biotic stimulus” and “response to external stimulus.” In order to study the classification features in GO terms in the two sugar‐mimic alkaloid treatments, we manually classified 46 GO terms related to resistance to host phytochemical defenses into major functional groups (Table 1). In these functional groups, GO terms in the D‐AB1 treatment were similar and specific to the 1‐DNJ treatment (Figure 2). GO terms related to immunity and stimulants were enriched in both D‐AB1 and 1‐DNJ treatments and were largely upregulated. Importantly, GO terms related to hydrolase activity, catabolic processes, metabolic processes, and oxidation‐reduction were only enriched in the D‐AB1 treatment. Two GO terms (associated with hydrolase activity and oxidation‐reduction) were overrepresented in genes exclusively downregulated in the D‐AB1 treatment, while the GO terms related to “metabolic” and “catabolic” varied in terms of the direction of the regulation (Figure 2). Nonetheless, *B. mori* seems to show a better adaptive performance in metabolism and catalysis during long‐term cross talk in its frequent encounters with the sugar‐mimic alkaloid (1‐DNJ). By contrast, the occasionally encountered D‐AB1 partly inhibits metabolic and physiological processes of *B. mori*.

**Table 1:** GO enrichment in response to sugar‐mimic alkaloids

#	GO. ID	GO term description	Genes	Enrichment	Genes	
				*p*‐Value	D‐AB1	1‐DNJ
Responses to immune						
1	GO:0002376	Immune system process	33	8.03E−06	13	11
2	GO:0006955	Immune response	32	5.34E−06	13	11
3	GO:0045087	Innate immune response	25	1.18E−05	11	11
4	GO:0009617	Response to bacterium	22	2.13E−05	10	12
5	GO:0042742	Defense response to bacterium	22	2.13E−05	10	12
Responses to stimulate						
6	GO:0006952	Defense response	33	4.72E−05	12	12
7	GO:0009605	Response to external stimulus	25	1.18E−05	11	12
8	GO:0009607	Response to biotic stimulus	23	3.43E−05	10	12
9	GO:0043207	Response to external biotic stimulus	23	3.43E−05	10	12
10	GO:0051707	Response to other organism	23	3.43E−05	10	12
11	GO:0098542	Defense response to other organism	23	3.43E−05	10	12
Extracellular						
12	GO:0005576	Extracellular region	181	4.61E−10	43	40
13	GO:0044421	Extracellular region part	68	2.97E−04	16	11
14	GO:0005615	Extracellular space	65	1.68E−04	16	11
Intracellular						
15	GO:0000785	Chromatin	75	2.78E−03	15	16
16	GO:0000786	Nucleosome	72	1.81E−03	15	16
17	GO:0032993	Protein‐DNA complex	72	1.81E−03	15	16
18	GO:0044815	DNA packaging complex	72	1.81E−03	15	16
Hydrolase activity						
19	GO:0017171	Serine hydrolase activity	48	3.87E−05	15	–
20	GO:0016798	Hydrolase activity, acting on glycosyl bonds	34	6.23E−05	12	–
21	GO:0004553	Hydrolase activity, hydrolyzing O‐glycosyl compounds	32	3.09E−05	12	–
Metabolic process						
22	GO:0005975	Carbohydrate metabolic process	63	3.58E−04	16	–
23	GO:0006022	Aminoglycan metabolic process	30	2.14E−03	9	–
24	GO:0006040	Amino sugar metabolic process	24	3.37E−04	9	–
25	GO:0006030	Chitin metabolic process	23	1.30E−03	8	–
26	GO:0006563	L‐serine metabolic process	3	1.05E−03	3	–
Catabolic process						
27	GO:1901136	Carbohydrate derivative catabolic process	15	2.40E−03	6	–
28	GO:0006026	Aminoglycan catabolic process	13	9.79E−04	6	–
29	GO:0006032	Chitin catabolic process	7	1.88E−04	5	–
30	GO:0046348	Amino sugar catabolic process	7	1.88E−04	5	–
31	GO:1901072	Glucosamine‐containing compound catabolic process	7	1.88E−04	5	–
Peptidase activity						
32	GO:0070011	Peptidase activity, acting on L‐amino acid peptides	120	3.34E−06	29	–
33	GO:0008236	Serine‐type peptidase activity	48	3.87E−05	15	–
34	GO:0004252	Serine‐type endopeptidase activity	39	2.80E−04	12	–
35	GO:0008237	Metallopeptidase activity	38	9.02E−04	11	–
36	GO:0008238	Exopeptidase activity	27	3.77E−06	12	–
37	GO:0004177	Aminopeptidase activity	14	1.50E−03	6	–
38	GO:0004180	Carboxypeptidase activity	13	9.36E−04	6	–
39	GO:0010951	Negative regulation of endopeptidase activity	6	1.65E−03	–	3
40	GO:0052548	Regulation of endopeptidase activity	6	1.65E−03	–	3
41	GO:0004866	Endopeptidase inhibitor activity	6	2.05E−03	–	3
42	GO:0004867	Serine‐type endopeptidase inhibitor activity	5	1.06E−03	–	3
Oxidation‐reduction						
43	GO:0016491	Oxidoreductase activity	261	8.69E−04	42	–
44	GO:0055114	Oxidation‐reduction process	252	4.22E−04	42	–
45	GO:0016705	Oxidoreductase activity, acting on paired donors,	95	3.29E−04	21	–
46	GO:0016717	Oxidoreductase activity, acting on paired donors,	14	1.50E−03	6	–
Not classified						
47	GO:0046914	Transition metal ion binding	201	1.19E−03	34	–
48	GO:0006508	Proteolysis	134	9.86E−05	28	–
49	GO:0046983	Protein dimerization activity	103	2.25E−05	–	16
50	GO:0006950	Response to stress	78	1.33E−04	–	12
51	GO:0044427	Chromosomal part	77	4.69E−06	–	16
52	GO:0046982	Protein heterodimerization activity	73	1.80E−07	–	16
53	GO:0044283	Small molecule biosynthetic process	50	1.03E−03	13	–
54	GO:0051704	Multiorganism process	49	8.35E−07	–	12
55	GO:0042302	Structural constituent of cuticle	41	1.80E−03	11	–
56	GO:0016053	Organic acid biosynthetic process	35	9.23E−05	12	–
57	GO:0046394	Carboxylic acid biosynthetic process	35	9.23E−05	12	–

Note

Significantly enriched GO terms in the set of differentially regulated genes fed on sugar‐mimic alkaloids were classified into major functional groups. The number and *p*‐value of gene annotated to each term that responded D‐AB1 are given and can be compared with those in 1‐DNJ.

### 3.3 D‐AB1 partly inhibits metabolic and physiological processes

**Figure 3:**
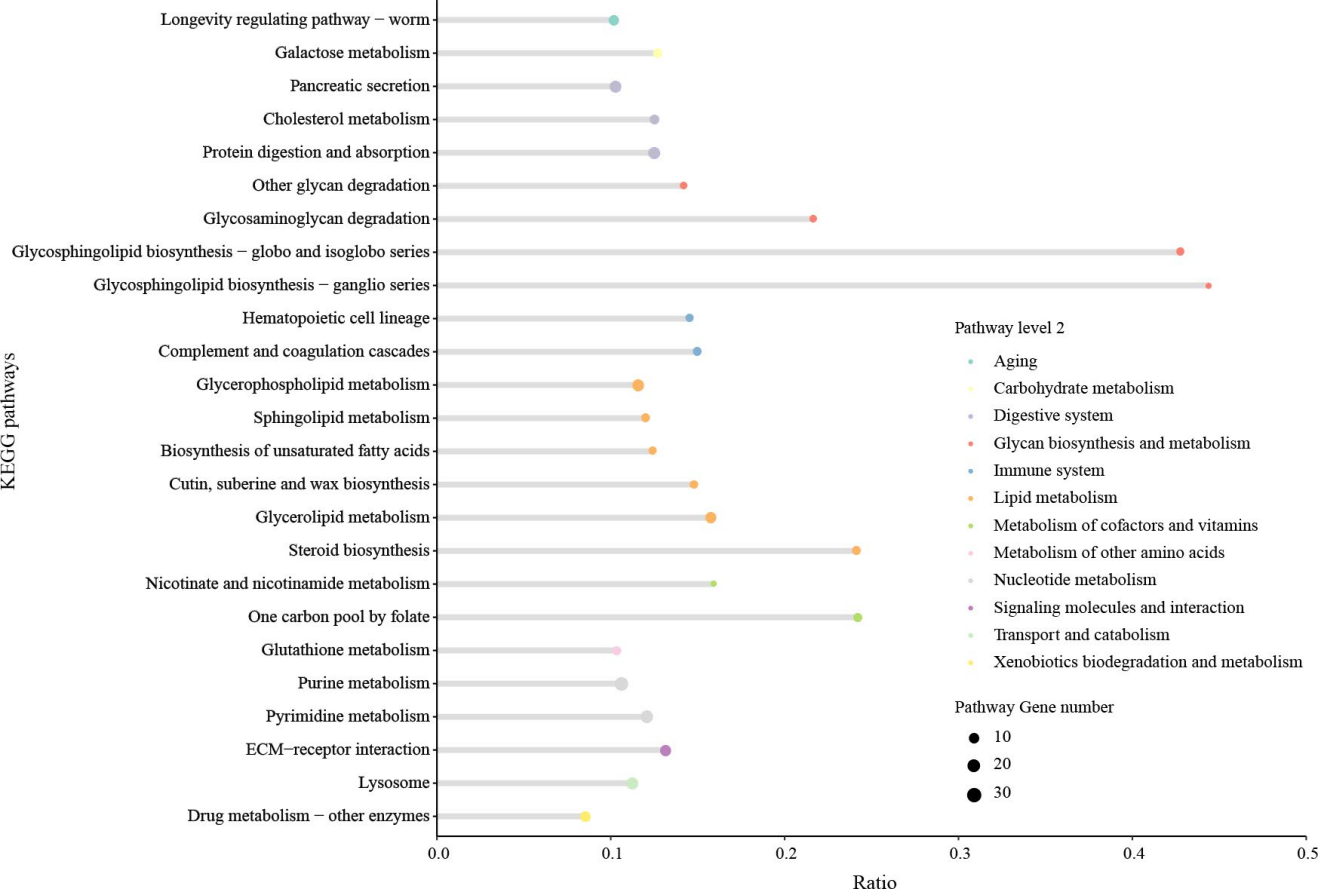
KEGG pathways of D‐AB1 specific DEGs. Different colors represent the level 2 of KEGG pathways. The size of point represents the numbers of enriched genes in pathways

To investigate the influence of metabolism pathways in occasionally encountered D‐AB1, we carried out KEGG enrichment analysis for D‐AB1 specific DEGs. For KEGG analysis, DEGs are mostly enriched in a variety of metabolism and defense pathways, like lipid metabolisms, glycan biosynthesis and metabolisms, and immune systems. In addition, several pathways related to digestive systems were also enriched (Figure 3). D‐AB1 had negative influence on sugar metabolism of insects (Asano et al., 2001; Diaz‐Lobo et al., 2016). As expected, the DEGs in glycosphingolipid biosynthesis pathways and other digestive pathways were mostly downregulated. D‐AB1 treatment also elicited many downregulated DEGs enriched in lipid metabolisms (Figure S4). As a consequence, the results of pathway analysis demonstrated that occasionally encountered D‐AB1 caused a substantial proportion of downregulated genes relating to carbohydrate metabolism, lipid metabolism, catabolic process, and glycan biosynthesis and metabolism.

### 3.4 Putative regulators in adaption of host plant chemicals

**Figure 4:**
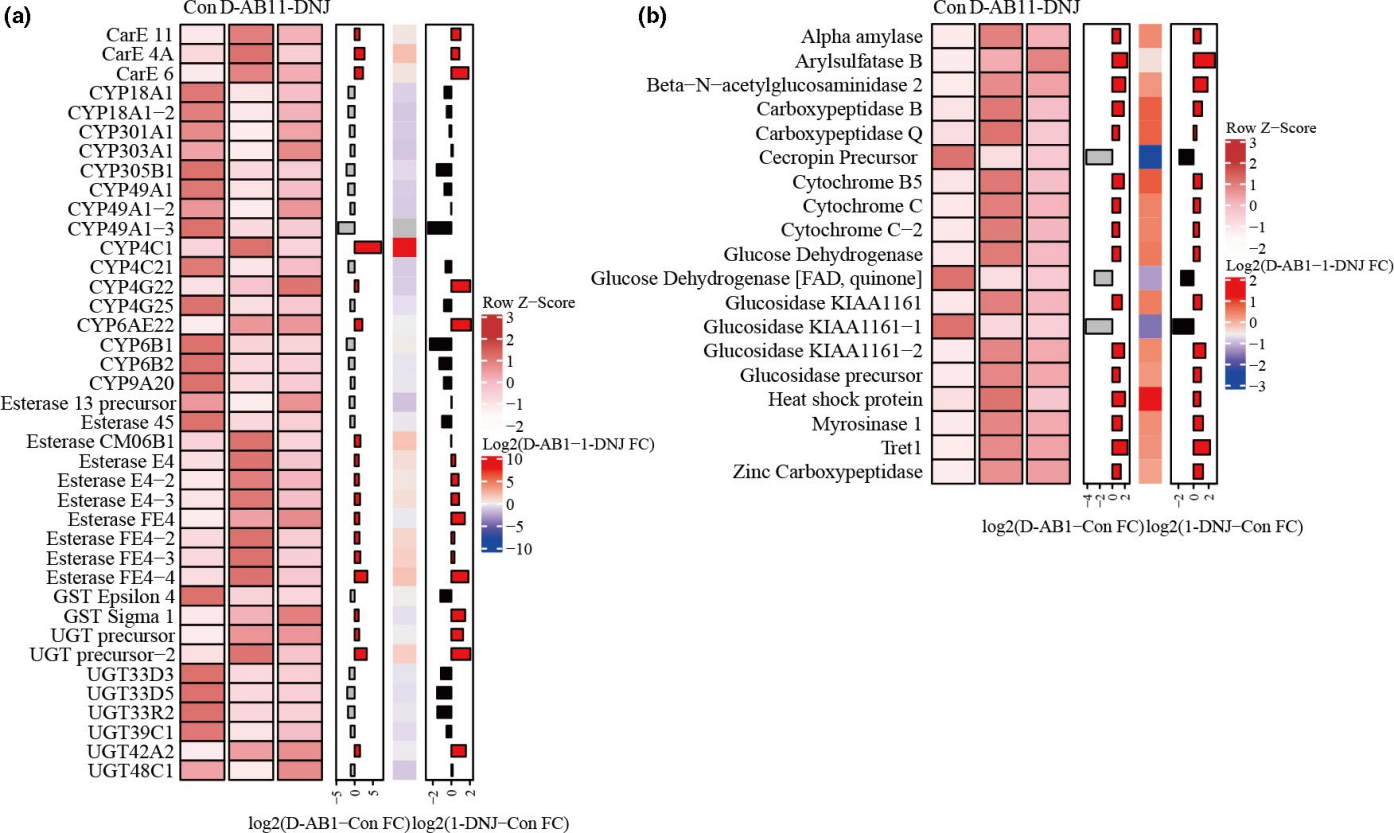
Transcriptional responses of *B. mori* to two sugar‐mimic alkaloids. (a) Heat map of putative detoxification genes significantly regulated in *B. mori* larvae feeding on both treatments. Heat map colors correspond to the scale value of FPKM. Row annotations are presented on bar plots and a single heat map, which display the fold change (FC) of treatment versus control or treatment versus treatment. (b) Heat map of genes associated with digestion and metabolism significantly regulated in *B. mori* larvae

Detoxification enzymes including esterases, cytochrome P450 monooxygenases (P450s), glutathione transferases (GSTs), carboxylesterases (CarEs), and UDP glycosy‐transferases (UGTs), appear to have a key role in plant–insect interactions (Birnbaum, Rinker, Gerardo, & Abbot, 2017; Peng, Li, Peng, Pei, & Zhou, 2018). Among the detoxification‐related DEGs, 39 genes are members of five gene families that are wildly known to be involved in detoxification processes and are associated with host plant utilization: 3 CarEs, 10 esterases, 2 GSTs, 16 P450s, and 8 UGTs (Table S4). P450 gene expression levels were typically regulated at a low level but that this changed in response to the appearance of sugar‐mimic alkaloids (Figure 4a). Our transcriptomes revealed that *CYP6AE22* and *CYP4G22* were significantly upregulated following each of the two sugar alkaloid treatments. UGTs, which act as catalysts for the transfer of a glycosyl group from UDP‐glucose to a variety of acceptor molecules, are implicated in insect resistance to plant chemicals (Mackenzie et al., 2005). Here, we found 8 DEGs with significant similarity to UGTs.

Apart from abovementioned general detoxification enzymes, we also verified a large number of differentially expressed genes associated with digestion as well as metabolism. Four glucosidase transcripts were differentially expressed after the D‐AB1 treatment (Figure 4b); three were upregulated, and one was downregulated. Two of abovementioned glucosidase were also exclusively upregulated in the 1‐DNJ treatment; however, the fold changes were lower when compared to the D‐AB1 treatment (Figure 4b). Additionally, the digestive enzyme, alpha amylase was upregulated after both of the sugar‐mimic alkaloid treatments.

Several upregulated transcripts played important roles in development and catalytic reactions, such as *trehalose transporter 1* (*Tret1*), *myrosinase 1*, and *arylsulfatase B* (Figure 4b). Sugar‐mimic alkaloids inhibit midgut hydrolysis activity thereby restraining trehalase uptake and trehalose utilization (Agrawal & Konno, 2009; Konno et al., 2006). Tret 1 mediates the bidirectional transfer of trehalose synthesized in the fat body (Ebner, Ritz, & Fumetti, 2019). In this study, *Tret1* was upregulated after both sugar‐mimic alkaloid treatments. We also observed that *myrosinase 1* and *arylsulfatase B* were upregulated after both sugar‐mimic alkaloid treatments (Figure 4b). Moreover, Aldo‐Keto Reductase (AKR2E4), plays an important role in 3‐dehydroecdysone conversion to ecdysone and spermatogenesis in silkworm testes (Yamamoto, Ozakiya, & Uno, 2017). *AKR2E4* was also upregulated after both types of sugar‐mimic alkaloid treatments. These results suggested that mulberry‐specialist insects evolved physiological adaptations in a multistep process, ensuring that fundamental metabolic processes were maintained.

### 3.5 Sugar‐mimic alkaloids caused specific time‐dependent changes in metabolic gene expression

**Figure 5:**
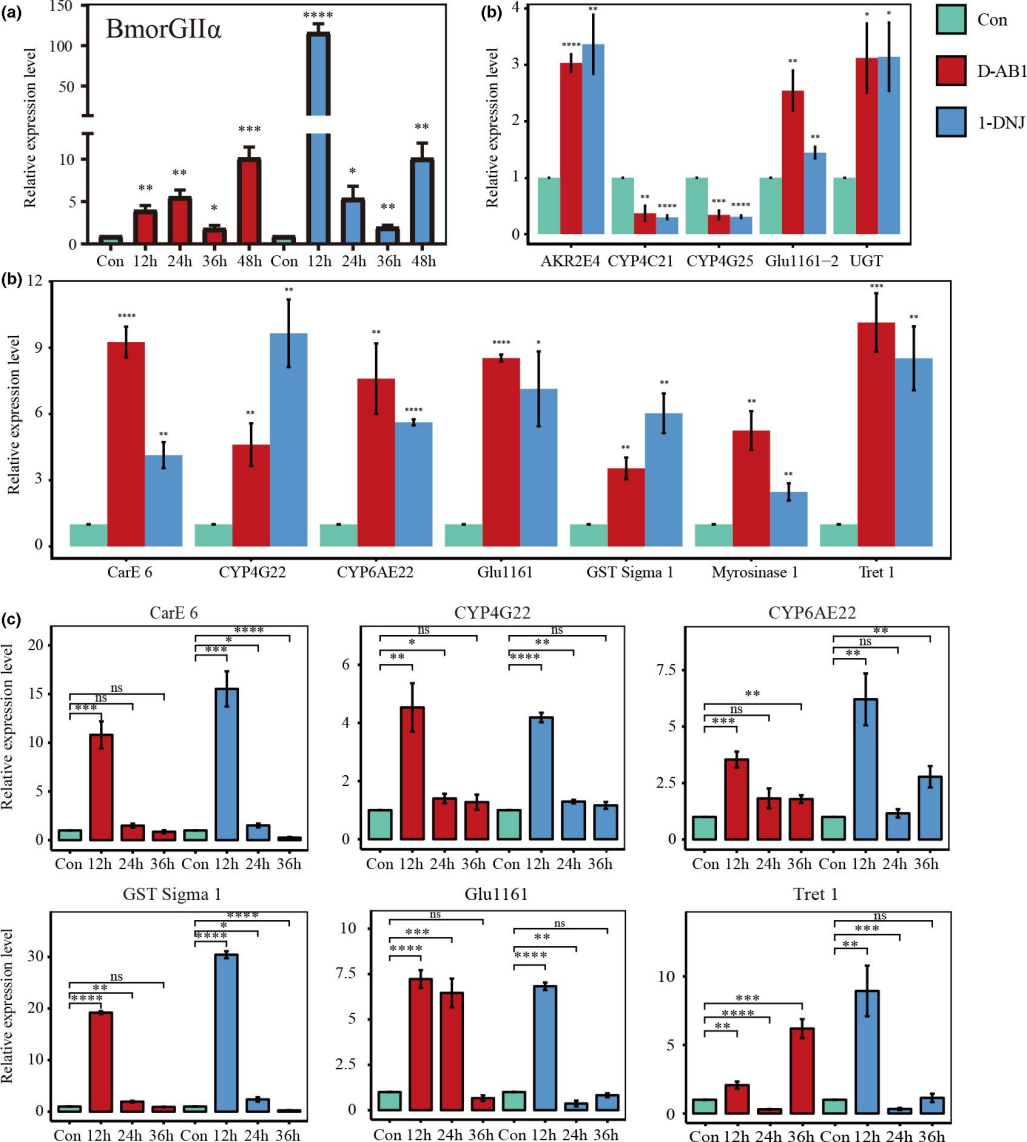
Quantitative RT‐PCR analysis of candidate gene expression of *B. mori* reared on sugar‐mimic alkaloids. (a) Temporal gradient qPCR of Gllα. (b) qRT‐PCR analysis of the expression level of putative candidate genes in transcriptome. (c) Temporal gradient qPCR of putative candidate genes. Graphs display the mean ± *SEM* fold difference in average mRNA level (four biological replicates of two treatments each; three technical replicates each). Bars and stars represent significant differences in gene expression when compared to untreated control (for statistical tests, a two‐tailed unpaired Student's *t* test was used throughout, n.s. nonsignificant; *p < .05; **p < .01; ***p < .0005; ****p < .0001)

The selected candidate genes were validated by qRT‐PCR. Our independent validation of differential expression was generally concordant with the transcriptome results (Figure 5). To determine whether changes in the level of gene expression after sugar‐mimic alkaloid treatment are time dependent, we designed temporal gradient qRT‐PCR assays. Glucosidase II is a type of nondigestive enzyme and GIIα is the main subunit to hydrolyze substrates (Okuyama et al., 2017). Our previous study suggested that the overexpression of *B. mori* GIIα was elicited to compensate for the inhibition of its activity by sugar‐mimic alkaloids (Li et al., 2018). Therefore, we first checked the expression of nondigestive glucosidase GIIα in the 1‐DNJ or D‐AB1 treatments after 12, 24, 36, and 48 hr (Figure 5a). Of all the time points examined during the two days of growth, GIIα was upregulated predominantly at 12 hr in the DNJ treatment, but the expression of GIIα was also upregulated at other time points generally. The transcript levels of the other 6 genes were also significantly induced after exposure to the sugar‐mimic alkaloid treatments at 12 hr, and most genes were increased again after 48 hr (Figure 5b,c). In conclusion, we found that D‐AB1 and 1‐DNJ caused specific time‐dependent changes in several metabolic gene expression, suggesting that they were involved in the adaption to the sugar‐mimic alkaloids.

## 4 DISCUSSION

In recent years, some general aspects of herbivory responses to plants are well resolved. However, the complex system‐wide processes that allow oligophagous herbivores to respond to host phytochemicals remain largely undetermined (Ffrench‐Constant, Daborn, & Le Goff, 2004; Onose et al., 2013). In this study, we thoroughly examined the transcriptomic response of a mulberry‐specialist insect to different sugar‐mimic alkaloids of the mulberry. We elucidated that *B. mori* developed multistep mechanisms including the employment of a detoxification enzyme system and the upregulation of the expression profile of metabolic‐related genes to coordinate adaptive responses and ensure optimal metabolism homeostasis. Furthermore, we analyzed the effects of plastic responses of *B. mori* to two different sugar‐mimics by characterizing the gene expression pattern and found that the expressions of several functionally important genes were significantly changed. *Bombyx mori* exhibits a similar and specific transcriptomic profile in response to two different sugar‐mimic alkaloids. The transcriptional responses of *B. mori* to often encountered 1‐DNJ showed a relative overlap with seldom encountered D‐AB1. On the other hand, D‐AB1 elicited a substantial proportion of downregulated genes relating to carbohydrate metabolism, catabolic process, lipid metabolism, and glycan biosynthesis and metabolism suggesting that this seldom encountered sugar‐mimic (D‐AB1) partly inhibits metabolic and physiological processes. These results substantially add novel insights into the adaptive mechanisms involved in plant–insect interactions.

Increasing the metabolic capability of detoxification systems and reducing phytochemical target site sensitivity have previously been reported in insects. Metabolic resistance is usually due to overproduction of “detoxifying enzymes” that metabolize heterogeneous organisms (Li, Schuler, & Berenbaum, 2007; Tan et al., 2019). Detoxification enzymes appear to have a key role in plant–insect interactions (Després et al., 2007). Here, the vast majority of differentially expressed transcripts involved in detoxification were found to be upregulated in *B. mori* larvae feeding on either of the two sugar‐mimic alkaloids. Such overrepresentation of GO terms associated with genes encoding monooxygenases and oxidoreductases indicated the initiation of a broad metabolic detoxification response when feeding on sugar‐mimic alkaloids. P450 metabolism of plant secondary metabolites plays a key role in plant–herbivore interactions (De Panis et al., 2016; Feyereisen, 2012). Previous study indicated that CYP6AE gene cluster has the role of detoxifying phytochemicals and insecticides in *Helicoverpa armigera* (Wang et al., 2018). The expression levels of *CYP6AE14* and *CYP6AB3* were produced by furanocoumarins and gossypol respectively in lepidopterans (Mao et al., 2007). Similarly, we identified that *CYP6AE22*, which clustered with the *CYP6AE* subfamily, was significantly increased after two sugar‐mimic alkaloids treatments. In addition, previous studies also showed that *CYP6AE22* was upregulated in silkworms fed with phoxim (Wang et al., 2013). Therefore, our findings, together with previous studies, suggest that *CYP6AE22* involved in the detoxification of phytochemicals. Recent studies also linked the *CYP4G* subfamily to insecticide resistance by means of thickening the cuticular barrier (Balabanidou et al., 2016; Yu et al., 2016). *CYP4G22* has an imidacloprid‐resistant effect in *Asian citrus psyllid* (Hemiptera: Lividae) (Tian, Li, Wang, Liu, & Zeng, 2018). Here, *CYP4G22* was significantly upregulated in *B. mori* after both sugar alkaloid treatments. Consequently, *CYP4G22* and *CYP6AE22* were elicited in concert due to chemical defense adaptations when ingesting sugar‐mimic alkaloids.

Glutathione transferases were originally grouped into three classes (I, II, and III) in insects and were also involved in the detoxification of various plant xenobiotics (Chelvanayagama, Parker, & Board, 2001; Enayati, Ranson, & Hemingway, 2005; Ranson & Hemingway, 2005; Tu & Akgül, 2005). Comparative proteomic analysis identified that GST‐Sigma 1 was positively correlated with the accumulation of 1‐DNJ in the silkworm (Chen et al., 2018; Flanagan & Smythe, 2011). We found that *GST‐sigma 1* and *GST‐epsilon 4* were significantly increased after either of two alkaloid treatments, suggesting both GSTs played a pivotal role in the interactions between *B. mori* and mulberry. The third important group of metabolic enzymes involved in insecticide resistance are carboxylesterases. CarEs are usually involved in insect resistance to carbamate and pyrethroid through gene amplification, upregulation, coding sequence mutation, or a combination of these mechanisms (Li et al., 2007). Our analysis clearly indicated that three CarEs including *CarE4A*, *CarE6*, and *CarE11* were significantly upregulated after D‐AB1 treatment and *CarE6* was significantly induced after 1‐DNJ treatment.

UDP glycosy‐transferases play a central role in the detoxification and elimination of a wide range of endogenous and exogenous compounds. The metabolism of plant compounds by UGTs has been reported in *Manduca. sexta,* and *B. mori* (Ahmad & Hopkins, 1993; Luque, Okano, & O'Reilly, 2002). In this study, we identified 9 DEGs that bore high similarity to UGTs. Among these, the UGT precursor (GeneID: 100500763) has 45% identity with BmUGT1, which is involved in the degradation of flavonoids and coumarins in *B. mori*. The majority of upregulated DEGs showed higher fold change in D‐AB1 than in the 1‐DNJ treatment. Taken together, these results suggest that the adaptation of phytophagous specialists to host sugar‐mimic alkaloids was a sequence of complex detoxification processes. The induction of detoxification enzymes might confer an adaptive plasticity to mulberry‐specialists that enables them to optimize their fitness in the presence of varying levels of sugar‐mimic alkaloids.

Plant chemicals are crucial defensive measures and exert influence on herbivores. In response, herbivorous insects have evolved a number of mechanisms to cope with these biotic stresses (Després et al., 2007; Li et al., 2007). Metabolic resistance is often associated with phenotypic plasticity, as the production of detoxification enzymes is usually induced by the presence of host plant chemicals. Here, we also found that several metabolic and digestive related genes underwent inordinate transcriptional changes to counter the chemical defense of host plants. Three glucosidases were significantly upregulated after D‐AB1 treatment, while two glucosidases were increased in the 1‐DNJ treatment (Figure 4b). These changes enabled *B. mori* to alleviate the effects of growth inhibition through regulation of the target enzymes. Sugar‐mimic alkaloids could impede digestion through their action on insect digestive alpha amylase (Franco, Rigden, Melo, & Grossi‐de‐Sá, 2002). The quantitative and qualitative adaptations of digestive α‐amylases are an important defense strategy against the inhibitors of the host plant (Pytelková et al., 2009). Here, *Alpha amylase* gene was also upregulated after both alkaloid treatments. Apart from the enzymes for detoxification and digestion, the adaptive plasticity of metabolic enzymes with other important physiological functions is a universal mechanism employed by herbivorous insects (Després et al., 2007; Heidel‐Fischer & Vogel, 2015; Li et al., 2007; Pytelková et al., 2009). In this study, several metabolic enzymes were also found to assist *B. mori* in maintaining homeostasis, such as Tret1, myrosinase 1, arylsulfatase B, and Aldo‐Keto Reductase. These significant changes in metabolic and digestive related genes revealed potential metabolic adaption to host phytochemicals in order to maintain the homeostasis of *B. mori*. Consistent with this observation, substantial alterations in the expression levels of transcripts involved in primary metabolism in response to different food environments have also been described in *Drosophila mettleri*, *Helicoverpa armigera*, and *Phaedon cochleariae* (Celorio‐Mancera et al., 2013; Hoang, Matzkin, & Bono, 2015; Müller et al., 2017).

In addition, we compared the effects of plastic responses of *B. mori* to occasionally encountered D‐AB1 and commonly encountered 1‐DNJ. We found that *B. mori* exhibits a similar and specific transcriptomic profile in response to two different sugar‐mimic alkaloids. 1‐DNJ caused only minor differences in gene expression profiles. In contrast, the majority of differentially expressed transcripts associated with hydrolase activity, catabolic processes, metabolic processes, and oxidation‐reduction displayed significant changes after D‐AB1 treatment. Many of the downregulated genes were related to lipid metabolism and glycan biosynthesis and metabolism. Most of intermediate metabolism occur in fat body, and lipid metabolism is an important physiological process and encoded the detoxification‐related genes (Jiang et al., 2020; Meng et al., 2019). These provoke numerous changes in the insect's physiology and evoke substantial plastic responses once the original host plant species is encountered again.

## 5 CONCLUSION

The adaptive evolution of chemical defense sequestration by herbivores is a highly complicated process requiring continued research efforts. Our work provided insight into genome‐wide transcriptional changes in relation to responses and adaptions of insect herbivores to two different sugar‐mimic alkaloids. Our results showed that the transcriptional plasticity in genes related to metabolism, detoxification, digestion, and general cellular processes played a key role in both long‐term adaptation and short‐term responses of the mulberry‐specialist insect to changing sugar‐mimic alkaloids. Two different sugar‐mimic alkaloids induced similar and specific insect defense responses. In addition, these results clearly indicated that a long‐term adaptive experience with 1‐DNJ caused the lowest changes in gene expression, and a low level of plastic responses. In stark contrast, the short‐term response to the seldom encountering D‐AB1 caused the largest changes in gene transcription. We elucidated that *B. mori* developed multistep mechanisms including the employment of a detoxification enzyme system and the upregulation of the expression profile of metabolic‐related genes to coordinate adaptive responses and ensure optimal metabolism homeostasis. These strategies are thought to facilitate mulberry‐specialist insects to survive on sugar‐mimic alkaloid‐rich foods in their ecological niche without incurring developmental disorders. Thus, our work facilitated a systematic understanding of the sequestration and adaptation of phytophagous specialists to the host defense system.

## CONFLICT OF INTEREST

The authors declare no conflict of interest.

## AUTHOR CONTRIBUTIONS

**Shunze Jia:** Conceptualization (equal); Data curation (equal); Formal analysis (equal); Methodology (equal); Project administration (equal); Resources (equal); Software (equal); Validation (equal); Writing‐original draft (equal). **Yinghui Li:** Conceptualization (equal); Data curation (equal); Formal analysis (equal); Methodology (equal); Project administration (equal); Validation (equal); Writing‐original draft (equal). **Xiangping Dai:** Conceptualization (equal); Data curation (equal); Formal analysis (equal); Project administration (equal); Validation (equal); Writing‐original draft (equal). **Xiaotong Li:** Conceptualization (equal); Data curation (equal); Methodology (equal); Project administration (equal); Software (equal); Writing‐original draft (equal). **Yanyan Zhou:** Conceptualization (equal); Data curation (equal); Formal analysis (equal); Project administration (equal); Software (equal); Writing‐original draft (equal). **Yusong Xu:** Conceptualization (equal); Data curation (equal); Formal analysis (equal); Funding acquisition (equal); Investigation (equal); Project administration (equal); Supervision (equal); Validation (equal); Writing‐original draft (equal). **Huabing Wang:** Conceptualization (equal); Data curation (equal); Formal analysis (equal); Funding acquisition (equal); Investigation (equal); Methodology (equal); Project administration (equal); Resources (equal); Writing‐original draft (equal); Writing‐review & editing (equal).

## Supporting information

Supplementary Material

## Data Availability

The raw data for qRT–PCR assay are accessible at the Dryad repository: https://doi.org/10.5061/dryad.44j0zpc9c. The data sets supporting the conclusions of this article are included within the Supporting information. Transcriptome data have been deposited into NCBI. BioProject ID is PRJNA634879. Accession IDs of Sequence Read Archive are SRR11841537–SRR11841545.
