# Supplementary material for: Physiological adaptations to sugar‐mimic alkaloids: Insights from Bombyx mori for long‐term adaption and short‐term response
Source: Ecol Evol. 2020 Aug 26;10(18):9682–95. doi: 10.1002/ece3.6574 (PMC7520222; doi:10.1002/ece3.6574)
Supplement: Supplementary file 1 — Supplementary Material [file ECE3-10-9682-s001.pdf]

Supplementary Information for:

Physiological adaptations to sugar-mimic alkaloids: Insights from *Bombyx mori* for long-term adaption and short-term response

Shunze Jia, Yinghui Li, Xiangping Dai, Xiaotong Li, Yanyan Zhou, Yusong Xu, Huabing Wang

College of Animal Sciences, Zhejiang University, Hangzhou 310058, China

Table of Contents:

**Table of Contents**	Page 1
**Table S1**	Page 2-3
**Table S2**	Page 4-8
**Table S3**	Page 9-11
**Table S4**	Page 12-13
**Figure S1**	Page 14
**Figure S2**	Page 15
**Figure S3**	Page 16
**Figure S4**	Page 17

Table S1. List of qPCR primers used in this paper

Primer	Sequence(5'to3')	Base number
BmorGIIα-F	ATACCCAAGATACAGGACGC	20
BmorGIIα-R	CTACTTCTCCATTCTGGTCC	20
AKR2E4-F	TCCATAGGCATCTCCAACTTTA	22
AKR2E4-R	GGGAGTGTACGCCATCACC	19
CYP4C21-F	TTATTGGGCTCGTACCCTGAG	21
CYP4C21-R	TCGTGGATGTTGAAGTTTATCG	22
CYP4G25-F	TGTCAAGGCGGCGGTTGTATG	21
CYP4G25-R	GCGAGGATCGTACAGAAAGACG	22
Glu1161-2F	CCACGGTCAGAATCAGTGTC	20
Glu1161-2R	CATACGAGTAGTTCGGGAGA	20
UGT-F	CTACCCAAGACTTCCCTAAAT	21
UGT-R	TTTGAATACTTGACACCCAGA	21
CarE6-F	GATGGTGCGGTTAGCGGAAAG	21
CarE6-R	CTCATCGTCTGGGTCATCTGT	21
CYP4G15-F	GTCGTGTTTCTAACAGACGCTAA	23
CYP4G15-R	ACAACGCTCTTGCTGTTCTCA	21
CYP6AE22-F	ACAATGTTCTTTACTGACCTGCT	23
CYP6AE22-R	AAGAAACGCACTGAGCAATCA	21
Glu1161-F	AAGGAGTTGCTGGTGAGGTGG	21
Glu1161-R	GATGGGAGGGTTCACGGGAGT	21
GST Sigma 1-F	TTCGGTCAGATGCCAGTATTA	21
GST Sigma 1-F	CAACTTCTTCAACATATCAGGGT	23

myrosinase1-F	CACAGTCGGCTTCGTATTG	19
myrosinase1-R	CGTATTCTGTCGTCGTCAAGTA	22
Tret1-F	GCCCTGGGATCGTTCCTT	18
Tret1-R	CCGAGTCTACGTCGTCTTGGTT	22

Table S2. GO terms of Control vs D-AB1

GO Term ID	GO Term	Level 1	Level 2	Term Candidate Gene Num	Total Candidate Gene Num	Term Gene Num	Total Gene Num	Rich Ratio	P value	Q value
GO:0005576	extracellular region	cellular_component	extracellular region	43	154	181	1655	0.2375691	4.61E-10	3.58E-07
GO:0008233	peptidase activity	molecular_function	catalytic activity	32	215	134	2124	0.238806	1.29E-06	0.0005013
GO:0008238	exopeptidase activity	molecular_function	catalytic activity	12	215	27	2124	0.4444444	3.77E-06	0.0005866
GO:0016787	hydrolase activity	molecular_function	catalytic activity	67	215	399	2124	0.1679198	2.66E-06	0.0005866
GO:0070011	peptidase activity, acting on L-amino acid peptides	molecular_function	catalytic activity	29	215	120	2124	0.2416667	3.34E-06	0.0005866
GO:0006955	immune response	biological_process	response to stimulus	13	184	32	1800	0.40625	5.34E-06	0.0006917
GO:0002376	immune system process	biological_process	immune system process	13	184	33	1800	0.3939394	8.03E-06	0.0007966
GO:0003824	catalytic activity	molecular_function	catalytic activity	135	215	1034	2124	0.1305609	8.20E-06	0.0007966
GO:0009605	response to external stimulus	biological_process	response to stimulus	11	184	25	1800	0.44	1.18E-05	0.000916
GO:0045087	innate immune response	biological_process	response to stimulus	11	184	25	1800	0.44	1.18E-05	0.000916

GO:0009617	response to bacterium	biological_process	multi-organism process	10	184	22	1800	0.4545455	2.13E-05	0.001377
GO:0042742	defense response to bacterium	biological_process	multi-organism process	10	184	22	1800	0.4545455	2.13E-05	0.001377
GO:0004553	hydrolase activity, hydrolyzing O-glycosyl compounds	molecular_function	catalytic activity	12	215	32	2124	0.375	3.09E-05	0.0015684
GO:0009607	response to biotic stimulus	biological_process	response to stimulus	10	184	23	1800	0.4347826	3.43E-05	0.0015684
GO:0043207	response to external biotic stimulus	biological_process	response to stimulus	10	184	23	1800	0.4347826	3.43E-05	0.0015684
GO:0051707	response to other organism	biological_process	multi-organism process	10	184	23	1800	0.4347826	3.43E-05	0.0015684
GO:0098542	defense response to other organism	biological_process	multi-organism process	10	184	23	1800	0.4347826	3.43E-05	0.0015684
GO:0008236	serine-type peptidase activity	molecular_function	catalytic activity	15	215	48	2124	0.3125	3.87E-05	0.0015833
GO:0017171	serine hydrolase activity	molecular_function	catalytic activity	15	215	48	2124	0.3125	3.87E-05	0.0015833
GO:0006952	defense response	biological_process	response to stimulus	12	184	33	1800	0.3636364	4.72E-05	0.0018355
GO:0016798	hydrolase activity, acting on glycosyl bonds	molecular_function	catalytic activity	12	215	34	2124	0.3529412	6.23E-05	0.0023051
GO:0016053	organic acid biosynthetic process	biological_process	cellular process	12	184	35	1800	0.3428571	9.23E-05	0.0031187

GO:0046394	carboxylic acid biosynthetic process	biological_process	cellular process	12	184	35	1800	0.3428571	9.23E-05	0.0031187
GO:0006508	proteolysis	biological_process	metabolic process	28	184	134	1800	0.2089552	9.86E-05	0.0031913
GO:0008152	metabolic process	biological_process	metabolic process	131	184	1055	1800	0.1241706	0.0001359	0.0042247
GO:0005615	extracellular space	cellular_component	extracellular region	16	154	65	1655	0.2461538	0.0001682	0.0050263
GO:0006032	chitin catabolic process	biological_process	cellular process	5	184	7	1800	0.7142857	0.0001876	0.005027
GO:0046348	amino sugar catabolic process	biological_process	metabolic process	5	184	7	1800	0.7142857	0.0001876	0.005027
GO:1901072	glucosamine-containing compound catabolic process	biological_process	metabolic process	5	184	7	1800	0.7142857	0.0001876	0.005027
GO:0004252	serine-type endopeptidase activity	molecular_function	catalytic activity	12	215	39	2124	0.3076923	0.00028	0.0072527
GO:0044421	extracellular region part	cellular_component	extracellular region	16	154	68	1655	0.2352941	0.000297	0.0074449
GO:0006040	amino sugar metabolic process	biological_process	metabolic process	9	184	24	1800	0.375	0.0003369	0.007699
GO:0016705	oxidoreductase activity, acting on paired donors, with incorporation or	molecular_function	catalytic activity	21	215	95	2124	0.2210526	0.0003288	0.007699

	reduction of molecular oxygen									
GO:1901071	glucosamine-containing compound metabolic process	biological_process	metabolic process	9	184	24	1800	0.375	0.0003369	0.007699
GO:0005975	carbohydrate metabolic process	biological_process	metabolic process	16	184	63	1800	0.2539683	0.0003583	0.007955
GO:0055114	oxidation-reduction process	biological_process	metabolic process	42	184	252	1800	0.1666667	0.0004218	0.0091036
GO:0016491	oxidoreductase activity	molecular_function	catalytic activity	42	215	261	2124	0.1609195	0.0008691	0.0182502
GO:0008237	metallopeptidase activity	molecular_function	catalytic activity	11	215	38	2124	0.2894737	0.0009021	0.0184449
GO:0004180	carboxypeptidase activity	molecular_function	catalytic activity	6	215	13	2124	0.4615385	0.0009363	0.0186533
GO:0006026	aminoglycan catabolic process	biological_process	metabolic process	6	184	13	1800	0.4615385	0.0009789	0.019015
GO:0044283	small molecule biosynthetic process	biological_process	metabolic process	13	184	50	1800	0.26	0.0010264	0.0194517
GO:0006563	L-serine metabolic process	biological_process	cellular process	3	184	3	1800	1	0.0010526	0.0194724
GO:0046914	transition metal ion binding	molecular_function	binding	34	215	201	2124	0.1691542	0.0011879	0.0214659
GO:0004563	beta-N-acetylhexosaminidase activity	molecular_function	catalytic activity	4	215	6	2124	0.6666667	0.0013011	0.0220108

GO:0006030	chitin metabolic process	biological_process	cellular process	8	184	23	1800	0.3478261	0.0013031	0.0220108
GO:0015929	hexosaminidase activity	molecular_function	catalytic activity	4	215	6	2124	0.6666667	0.0013011	0.0220108
GO:0004177	aminopeptidase activity	molecular_function	catalytic activity	6	215	14	2124	0.4285714	0.0015015	0.0243049
GO:0016717	oxidoreductase activity, acting on paired donors	molecular_function	catalytic activity	6	215	14	2124	0.4285714	0.0015015	0.0243049
GO:0000786	nucleosome	cellular_component	organelle part	15	154	72	1655	0.2083333	0.0018063	0.0269901
GO:0032993	protein-DNA complex	cellular_component	macromolecular complex	15	154	72	1655	0.2083333	0.0018063	0.0269901
GO:0042302	structural constituent of cuticle	molecular_function	structural molecule activity	11	215	41	2124	0.2682927	0.0017995	0.0269901
GO:0044815	DNA packaging complex	cellular_component	macromolecular complex	15	154	72	1655	0.2083333	0.0018063	0.0269901
GO:0006022	aminoglycan metabolic process	biological_process	metabolic process	9	184	30	1800	0.3	0.0021353	0.0313048
GO:1901136	carbohydrate derivative catabolic process	biological_process	metabolic process	6	184	15	1800	0.4	0.0023954	0.0344671
GO:0000785	chromatin	cellular_component	organelle part	15	154	75	1655	0.2	0.0027764	0.0392236

Table S3. Go terms of Control vs 1-DNJ

GO Term ID	GO Term	Level 1	Level 2	Term Candidate Gene Num	Total Candidate Gene Num	Term Gene Num	Total Gene Num	Rich Ratio	P value	Q value
GO:0005576	extracellular region	cellular_component	extracellular region	40	98	181	1655	0.2209945	1.41E-15	7.11E-13
GO:0009607	response to biotic stimulus	biological_process	response to stimulus	12	82	23	1800	0.5217391	3.21E-11	2.31E-09
GO:0009617	response to bacterium	biological_process	multi-organism process	12	82	22	1800	0.5454545	1.59E-11	2.31E-09
GO:0042742	defense response to bacterium	biological_process	multi-organism process	12	82	22	1800	0.5454545	1.59E-11	2.31E-09
GO:0043207	response to external biotic stimulus	biological_process	response to stimulus	12	82	23	1800	0.5217391	3.21E-11	2.31E-09
GO:0051707	response to other organism	biological_process	multi-organism process	12	82	23	1800	0.5217391	3.21E-11	2.31E-09
GO:0098542	defense response to other organism	biological_process	multi-organism process	12	82	23	1800	0.5217391	3.21E-11	2.31E-09
GO:0009605	response to external stimulus	biological_process	response to stimulus	12	82	25	1800	0.48	1.15E-10	7.23E-09
GO:0045087	innate immune response	biological_process	response to stimulus	11	82	25	1800	0.44	2.38E-09	1.33E-07
GO:0006952	defense response	biological_process	response to stimulus	12	82	33	1800	0.3636364	5.82E-09	2.94E-07
GO:0006955	immune response	biological_process	response to stimulus	11	82	32	1800	0.34375	5.30E-08	2.43E-06
GO:0002376	immune system process	biological_process	immune system process	11	82	33	1800	0.3333333	7.66E-08	3.23E-06

GO:0046982	protein heterodimerization activity	molecular_function	binding	16	104	73	2124	0.2191781	1.80E-07	7.00E-06
GO:0051704	multi-organism process	biological_process	multi-organism process	12	82	49	1800	0.244898	8.35E-07	3.01E-05
GO:0000786	nucleosome	cellular_component	organelle part	16	98	72	1655	0.2222222	1.81E-06	5.37E-05
GO:0032993	protein-DNA complex	cellular_component	macromolecular complex	16	98	72	1655	0.2222222	1.81E-06	5.37E-05
GO:0044815	DNA packaging complex	cellular_component	macromolecular complex	16	98	72	1655	0.2222222	1.81E-06	5.37E-05
GO:0000785	chromatin	cellular_component	organelle part	16	98	75	1655	0.2133333	3.24E-06	9.08E-05
GO:0044427	chromosomal part	cellular_component	organelle part	16	98	77	1655	0.2077922	4.69E-06	0.0001246
GO:0005694	chromosome	cellular_component	cell	16	98	78	1655	0.2051282	5.61E-06	0.0001418
GO:0046983	protein dimerization activity	molecular_function	binding	16	104	103	2124	0.1553398	2.25E-05	0.0005415
GO:0006950	response to stress	biological_process	response to stimulus	12	82	78	1800	0.1538462	0.0001333	0.0030596
GO:0004867	serine-type endopeptidase inhibitor activity	molecular_function	molecular function regulator	3	104	5	2124	0.6	0.0010619	0.0230377
GO:0005615	extracellular space	cellular_component	extracellular region	11	98	65	1655	0.1692308	0.0010949	0.0230377
GO:0010951	negative regulation of endopeptidase activity	biological_process	biological regulation	3	82	6	1800	0.5	0.0016509	0.0308782
GO:0044421	extracellular region part	cellular_component	extracellular region	11	98	68	1655	0.1617647	0.0016139	0.0308782
GO:0052548	regulation of endopeptidase activity	biological_process	biological regulation	3	82	6	1800	0.5	0.0016509	0.0308782

GO:0004866	endopeptidase inhibitor activity	molecular_function	molecular function regulator	3	104	6	2124	0.5	0.0020482	0.0345275
GO:0046777	protein autophosphorylation	biological_process	cellular process	2	82	2	1800	1	0.0020511	0.0345275
GO:0061135	endopeptidase regulator activity	molecular_function	molecular function regulator	3	104	6	2124	0.5	0.0020482	0.0345275
GO:0043548	phosphatidylinositol 3-kinase binding	molecular_function	binding	2	104	2	2124	1	0.0023756	0.0374893
GO:0043560	insulin receptor substrate binding	molecular_function	binding	2	104	2	2124	1	0.0023756	0.0374893

Table S4. Gene families that involved in detoxification processes and related to host plant utilization

Description	Gene ID	log2(DAB/Control)	log2(DNJ/Control)
CarE 11	100144573	1.365784	0.987534
CarE 4A	101741370	2.699932	0.860294
CarE 6	101746653	2.281175	1.845883
CYP18A1	100036578	-1.71624	-0.75355
CYP18A1-2	101740852	-1.73645	-0.49992
CYP301A1	101737332	-1.32971	-0.21308
CYP303A1	101743283	-1.13128	0.158573
CYP305B1	100127103	-2.22188	-1.58738
CYP49A1	101736880	-1.79212	-0.75072
CYP49A1-2	101737184	-1.16166	-0.0272
CYP49A1-3	105842283	-4.47968	-2.52325
CYP4C1	101743022	7.276762	
CYP4C21	101738326	-1.71582	-0.63417
CYP4G22	101745427	1.023954	2.032293
CYP4G25	100127117	-1.19174	-0.79853
CYP6AE22	100126549	2.103136	2.106106
CYP6B1	101736314	-2.17505	-2.31549
CYP6B2	101735937	-1.59258	-1.30108
CYP9A20	100036579	-1.08483	-0.80823
Esterase 13 precursor	692864	-1.37933	0.030818
Esterase 45	100126869	-1.1945	-1.00263
Esterase CM06B1	101738914	1.594128	-0.03211
Esterase E4	101735768	1.12683	0.423168
Esterase E4-2	101739239	1.171617	0.776451
Esterase E4-3	101739374	1.476231	0.825803
Esterase FE4	101737676	1.276872	1.446148
Esterase FE4-2	101737808	1.320841	0.326892
Esterase FE4-3	101738777	1.558807	0.326582
Esterase FE4-4	101740499	3.477386	1.834146
GST Epsilon 4	100141440	-1.10912	-1.1954
GST Sigma 1	692631	1.094198	1.486239

UGT precursor	100500755	1.240582	1.258109
UGT precursor-2	100500763	3.315265	2.048655
UGT33D3	100862803	-1.42193	-1.12688
UGT33D5	100862845	-2.0316	-1.53922
UGT33R2	100862842	-1.805	-1.52
UGT39C1	100862811	-1.09879	-0.52793
UGT42A2	100862818	1.465181	1.561785
UGT48C1	100862824	-1.07289	0.141691

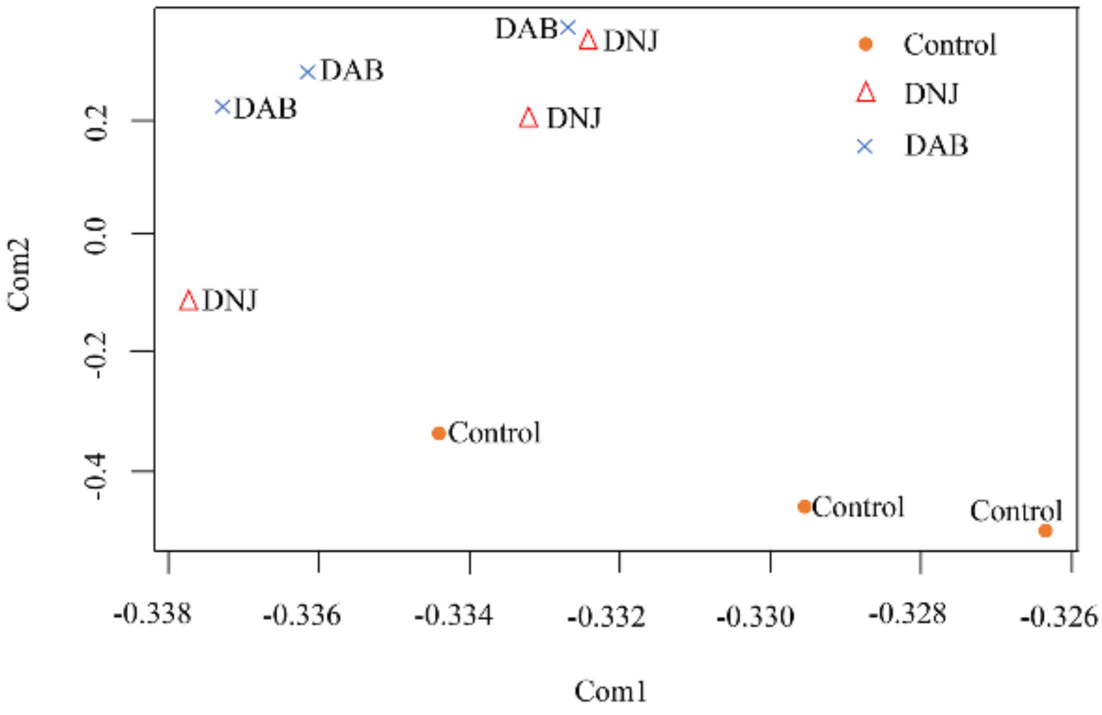

Figure S1. PCA analysis of transcriptome

PCA analysis was constructed based on expression value of transcriptome by princomp () in R.

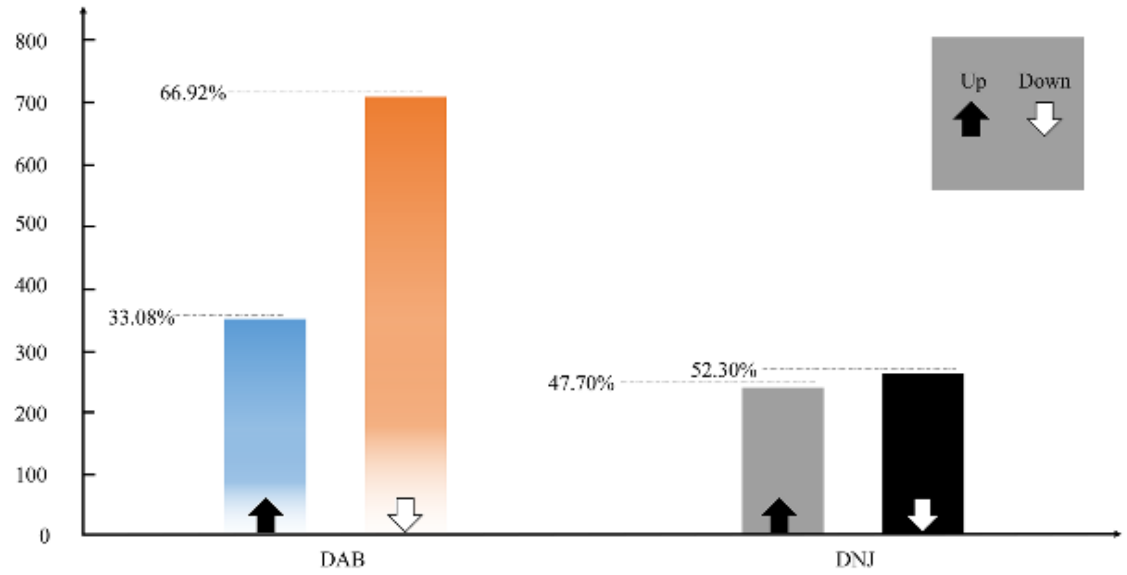

Figure S2. Expression level of D-AB1 and 1-DNJ treatment

The expression level of DEGs in two alkaloids treatments. Black arrowhead represented upregulation. White arrowhead represented downregulation.

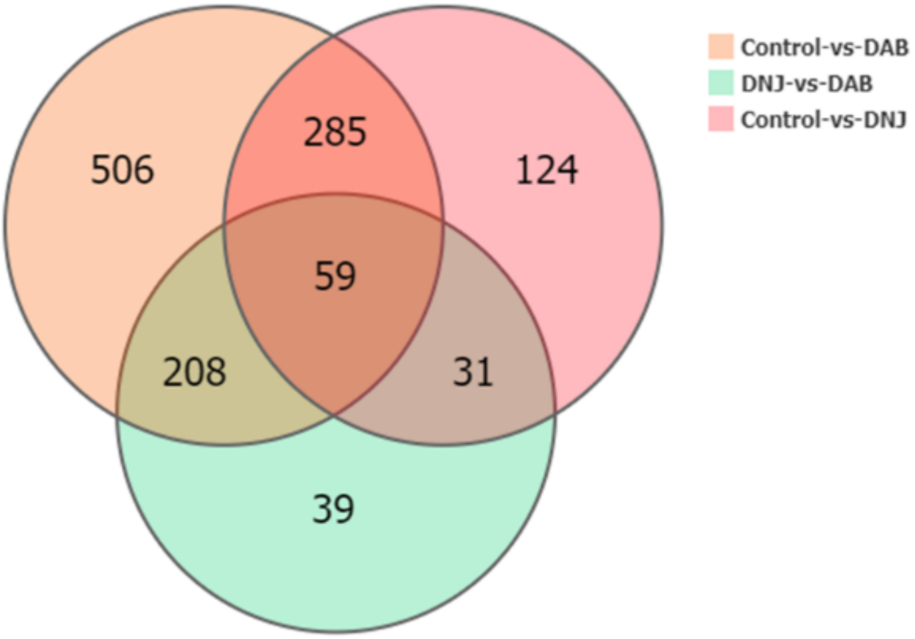

Figure S3. Venn diagram of different treatments

Venn diagram showing the overlap of differentially regulated genes in D-AB1 treatment with genes treated by 1-DNJ.

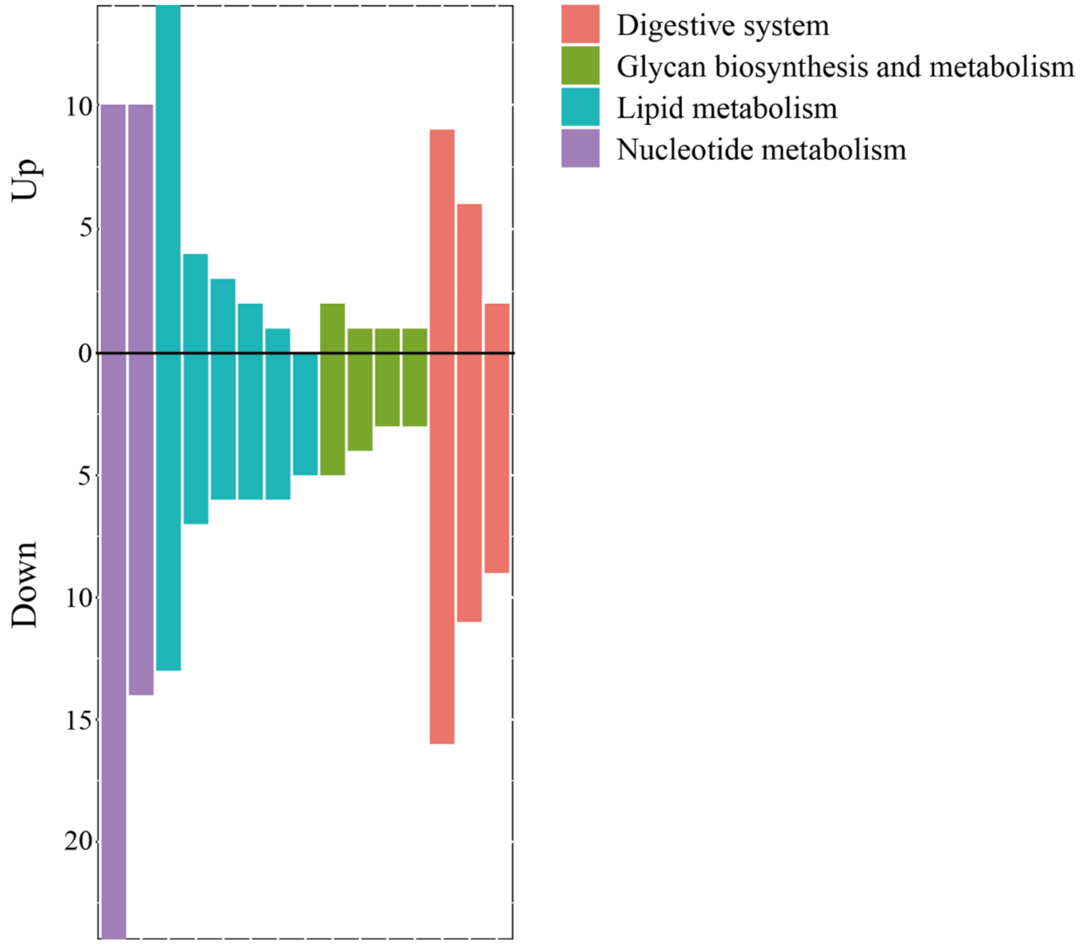

Figure S4. Number of up and down regulated DEGs within detoxification and metabolism related pathways

The colour indicated the level 2 of related pathways. A single bar represented a single pathway. And the number are numbers of significantly upregulated or significantly downregulated DEGs in single pathway.
